# Acute and Subacute Toxicity Study of Essential Oil of Cymbopogon Martini in Mice

**DOI:** 10.1155/2022/1995578

**Published:** 2022-12-17

**Authors:** Kassahun Dires Ayenew, Yihenew Sewale, Yosef Eshetie Amare, Amare Ayalew

**Affiliations:** ^1^Asrat Woldeyes Health Science Campus, Debre Berhan University, Debre Berhan, P.O. Box 445, Ethiopia; ^2^College of Natural and Computational Science, Debre Berhan University, Debre Berhan, P.O. Box 445, Ethiopia

## Abstract

**Background:**

Local Ethiopians regularly use *Cymbopogon martini* for cosmetic purposes. The plant's safety, however, is not supported by any solid facts. This investigation aimed to evaluate the acute and subacute toxicities of *C. martini* essential oil in mice.

**Methods:**

The essential oil was analyzed using GC-MS. The approach outlined by Chinedu et al., 2013 has been used to calculate the median lethal dose. According to organization for economic cooperation and development (OECD) 407 standard, a 28-day repeated dose oral toxicity study was carried out on female mice. Three groups of ten experimental mice each were distributed at random. Group I received the same saline volume and was considered the control. Groups II and III were treated with doses of *C. martini* of 500 mg/kg and 1000 mg/kg, respectively, of body weight. Hematological and biochemical markers were assessed. The liver and kidney were taken out after the sacrifice using sodium pentobarbital for pathological analysis.

**Results:**

Geraniol (40.89%) was the predominant component in the essential oil composition of *C. martini* with cyclofenchene (13.91%), myrcene (9.34%), 2, 4, 6, octatriene, 2, 6, dimethyl (8.20%), and ocimene (5.93%) being present in small amounts. The LD_50_ of *C. martini* essential oil was discovered to be greater than 5000 mg/kg body weight. During a 4-week follow-up period, mice treated with *C. martini*, the essential oil, at doses of 500 mg/kg or 1000 mg/kg body weight showed no evidence of toxicity or mortality. Biochemical and hematological parameters were not significantly altered in mice treated with the essential oil of *C. martini* compared with the control group. Histopathological evaluation of the liver and kidney did not exhibit any adverse results.

**Conclusions:**

The essential oil of *C. martini* from Ethiopia is considered relatively safe and nontoxic.

## 1. Introduction

Herbal remedies have been used extensively to cure illnesses and as a source of contemporary medications [1]. Around 80% of the world's population heavily relies on herbal medicine, and its use is expanding globally [2, 3]. Due to the various limitations of contemporary medications, attention is being paid to herbal remedies.

About 70% of the active components used today or in clinical trials are derived from natural sources, making them a rich source of biodiversity for identifying pharmaceutical lead compounds and developing new drugs [4, 5]. Not only do pharmacodynamic and pharmacokinetic parameters need to be known but also the drug's safety [6].

The primary reasons for discontinuing medications from clinical studies are hepatotoxicity and nephrotoxicity [7, 8]. Aminotransferases, alkaline phosphatase, sorbitol dehydrogenase, glutamate dehydrogenase, gamma-glutamyl-transferase, total bilirubin, total bile acids, and 5′-nucleotidase are some of the common biomarkers used to assess the safety of drugs for hepatotoxicity [9].

A significant volume of blood in circulation (approximately 25% of cardiac output) may be related to the kidneys' high susceptibility to active substances or their metabolites. More significantly, the concentration of toxins in the blood in the renal tubular fluid is linked to the role of nephrons in the processes of urine generation [7]. Blood urea nitrogen and serum creatinine in experimental animals have been suggested as markers for nephrotoxicity assessment [10].

90% of the population in Ethiopia relies on traditional medicine [11]. Local Ethiopians utilize *Cymbopogon martini* for cosmetic purposes.

*C. martini* belongs to the family of Poaceae. It is commonly called palmarosa which has historically been used as a culinary ingredient and for fumigation. Essential oils from *C. martini* have been shown to have cytotoxic, antibacterial, and antioxidant effects [12].

Despite being widely regarded as safe medications with a high therapeutic index, medicinal plants naturally contain metabolites that have the potential to harm humans, animals, and the environment [13].

*C. martini* ethnopharmacology has been documented. However, studies on safety profiles are lacking. This study aimed to examine the safety of *C. martini* essential oil in mice.

## 2. Materials and Methods

### 2.1. Plant Material Collection and Identification

Fresh leaves of *C. martini* were collected from the botanical garden of the Debre Berhan University-Ankober Herbal Medicine Research Center at Chefa Kebele in the North Shewa zone of the Amhara region, Ethiopia. The Botanist identified and authenticated the leaves in the Botanical Garden of Debre Berhan University, Ethiopia. For future use, voucher specimen No. CM 2012 was deposited in the herbarium of Addis Ababa University, Addis Ababa, Ethiopia.

### 2.2. Method of Extraction

The leaves were steam distilled to produce *C. martini* essential oil. In order to ensure that the steam was delivered properly, the plant materials were placed into the still and stacked in layers. This approach was centered on putting the fragrant plant via steam. The plant's volatile substances were collected by steam, which washed them away after passing through a serpentine that was chilled with cold water. A separator funnel was used to separate the distillate which contained a mixture of hydrosol vapor and essential oil. After drying over sodium sulfate, the essential oil was put into clean brown glass bottles to be kept until required [14].

### 2.3. Phytochemical Analysis

GC-MS determined the phytochemical analysis of the essential oil composition of *C. martini* according to the method described previously [15, 16].

### 2.4. Experimental Animals

In this investigation, three-month-old mice were employed. They were obtained from the pharmacology department of Addis Ababa University, Addis Ababa, Ethiopia. Mice ranged in weight from 25 to 35 grams. They were given regular pellets to eat, and unlimited tap water was available. The procedure involved keeping mice in a sanitary cage at ambient temperature [17]. The institutional review board of Debre Berhan University in Debre Berhan, Ethiopia, granted ethical approval for the use of experimental mice. Mice were treated and handled in compliance with ethical standards adopted globally.

### 2.5. Determination of the Median Lethal Dose (LD_50_)

A limit dose of 5000 mg/kg body weight was used to determine the LD_50_ of the essential oil of *C. martini*. The LD_50_ was calculated according to the new approach outlined by Chinedu et al., 2013 [18]. Four female mice were employed in the study's early phase. There were a total of four groups, each with one mouse. *C. martini* was administered in doses of 10 mg/kg and 100 mg/kg body weight (b.w) to Groups I and II, respectively. Groups III and IV were treated with 300 mg/kg and 600 mg/kg b.w., respectively. Mice were observed for an hour after the administration, then for ten minutes every two hours for the next 24 hours. In the second step, three female mice were used. They were randomly allocated into three groups. The doses given to Groups I and II were 1000 mg/kg and 1500 mg/kg b.w. Group III received treatment with 2000 mg/kg b.w. of *C. martini* essential oil. After treatment, animals were observed.

In the last round of LD_50_ determination, three female mice were also used. The placement of the animals into three groups of one was random. The essential oil of *C. martini* was administered at doses of 3000 mg/kg, 4000 mg/kg, and 5000 mg/kg b.w. to Groups I, II, and III, respectively. Following administration, meticulous observation was carried out for one hour, then for ten minutes every two hours for the following twenty-four hours. Two mice were used in a confirmatory test at 5000 mg/kg b.w.

### 2.6. Sub Acute Toxicity Study

According to the Organization for Economic Cooperation and Development's (OECD) 407 standard, the repeated dose 28-day oral toxicity study was carried out on female mice [19]. Since the responses are usually similar, the available literature suggests that any sex is used for subacute toxicity. When differences do exist, however, female mice are generally more sensitive [20, 21]. Three groups of ten experimental mice were randomly assigned into three groups of 10 animals each. The exact amount of saline was administered daily to Group I as the control group. The doses of 500 mg/kg and 1000 mg/kg b.w. of *C. martini* essential oil were given once daily to Groups II and III, respectively. These doses were established based on the findings of acute toxicity and the limit dose concept described by the European Chemical Agency in 2022 [22].

The animals were observed daily for toxicity, death, abnormal behavior, and other signs throughout the trial. Clinical observations were gathered and recorded prior to the test chemical's distribution and each day after. The number of mice in each cage, their weights, and how much food they ate each week were recorded. On the 28^th^ day, the only thing mice were allowed to drink was water. The next morning, mice were weighed, and blood was taken from a common carotid artery for hematological and biochemical examinations. Sodium pentobarbital was used to finish the experiment by killing all mice from both experimental groups.

Blood was extracted into a precalibrated tube containing EDTA for hematological assays. Hematological parameters including mean cell hemoglobin (MCH), mean cell volume (MCV), mean cell hemoglobin concentration (MCHC), hemoglobin (HB), hematocrit (HCT), and platelet count (PC) were analyzed using a hematological analyzer. For biochemical examination, the serum was separated, and samples were centrifuged at 3000 rpm for 10 minutes. Aspartate transaminase (AST), alanine transaminase (ALT), alkaline phosphate (ALP), total cholesterol (TC), blood urea nitrogen (BUN), and creatinine (CREA) were measured using an automated chemical analyzer. All animals had their livers and kidneys removed for gross anatomical and histological examinations. Formalin buffered at 10% was used to preserve the organs. Hematoxylin and eosin were used to stain the tissues, which were subsequently sliced to a thickness of 5 *μ*m, placed on glass slides, and examined under a light microscope. Photomicro-graphs were taken at 4 and 40 times [23].

### 2.7. Statistical Analysis

Results are presented in terms of the mean and standard errors of the mean (SEM). A one-way analysis of variance (ANOVA) and the Bonferroni multiple comparison tests were used to assess the data. At *P* < 0.05, differences were deemed statistically significant.

## 3. Results

The mice used in the experiment were all healthy. In the acute toxicity investigation, 12 female mice in total were employed. In subacute toxicity investigations, 30 female mice were used, and every animal followed the prescribed treatment plan.

### 3.1. Phytochemical Analysis

Geranial (40.89%) was the predominant component in the essential oil composition of *C. martini* with cyclofenchene (13.91%), myrcene (9.34%), 2, 4, 6, octatriene, 2, 6, dimethyl (8.20%), and ocimene (5.93%) being present in small amounts. Furthermore, there were trace amounts of hexadecanoic acid, bisabolone, 11-octadecenoic acid, methyl stearate, 4- undeccanone, citral, citronellol, and caryophyllene.

### 3.2. Effect on Acute Toxicity

Mice receiving oral doses of the essential oils of *C. martini* up to 5000 mg/kg did not exhibit treatment-related mortality, and no symptoms of toxicity or morbidity were noticed. The LD_50_ of *C. martini* essential oils was found to be greater than 5000 mg/kg b.w.

### 3.3. Effect on Subacute Toxicity

During a 4-week follow-up period, female mice treated with 500 mg/kg and 1000 mg/kg b.w. of the essential oils of *C. martini* showed no evidence of toxicity or death due to the treatment. As can be observed in Table 1, there were no appreciable variations in body weight between the mice treated with *C. martini* essential oils at doses of 500 mg/kg and 1000 mg/kg b.w. and the control group.

When compared to mice that received normal saline, mice were treated with essential oils of *C. martini* at doses of 500 mg/kg and 1000 mg/kg b.w. did not consume food in significantly different ways, as shown in Table 2.

No appreciable variations were seen between mice given *C. martini* essential oil and the control group's hematological parameters, which were all within the normal range (Table 3).

As shown in Table 4, compared to the control group, subacute administration of *C. martini* essential oil in mice at doses of 500 mg/kg and 1000 mg/kg b.w. exhibited no significant changes in all biochemical markers assessed.

### 3.4. Effect on the Histology of the Liver and Kidney

**Figure 1:**
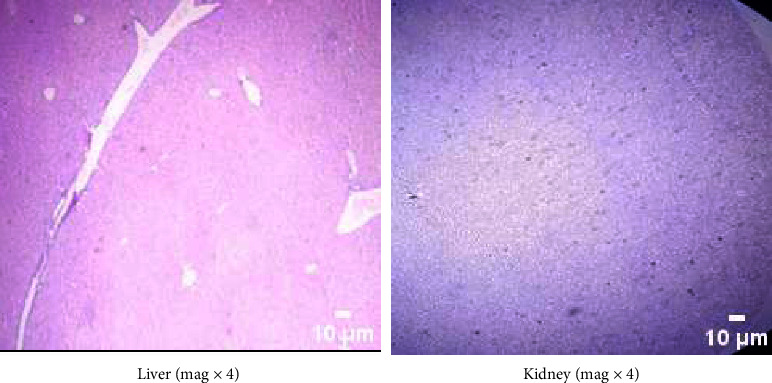
Histology of the liver and kidneys of control mice. The liver and kidney showed no histological changes. (a) Liver (mag x4). (b) Kidney (mag x4).

**Figure 2:**
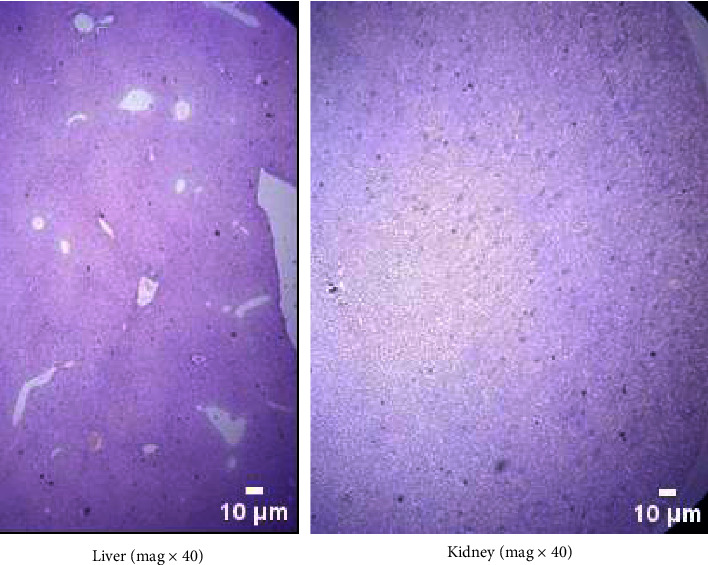
Histology of the liver and the kidney of mice treated at a dose of 500 mg/kg indicating normal liver and kidney cells. (a) Liver (mag x40). (b) Kidney (mag x40).

**Figure 3:**
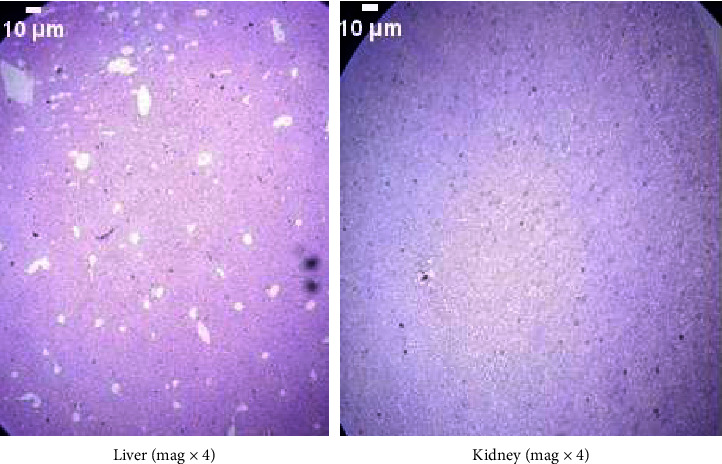
Histology of the liver and kidney of mice administered with *C. martini* essential oil at 1000 mg/kg demonstrating the lack of abnormalities in the liver and kidney cells. (a) Liver (mag x4). (b) Kidney (mag x4).

The liver and kidneys of the control mice were healthy, as seen in Figure 1. Both the cellular topologies and the integrity of the cells were unaffected when comparing the two tissues in the experimental groups to their respective controls. Figures 2 and 3 show that neither the kidneys nor the liver of the experimental animals showed any histological abnormalities.

## 4. Discussion

Cosmetics sectors all utilize essential oils often. The toxicity of these products is becoming a topic of growing study [24]. The primary reasons for discontinuing effective medications from clinical studies are hepatotoxicity and nephrotoxicity [7, 8].

In the current study, geraniol (40.89%) was the predominant component in the essential oil composition of *C. martini* with cyclofenchene (13.91%), myrcene (9.34%), 2, 4, 6, octatriene, 2, 6, dimethyl (8.20%), and ocimene (5.93%) being present in small amounts. Furthermore, there were trace amounts of hexadecanoic acid, bisabolone, 11-octadecenoic acid, methyl stearate, 4- undeccanone, citral, citronellol, and caryophyllene. A previous study on phytochemical analysis of *C. martini* reported that geraniol was the primary compound (57.49%) followed by geranyl acetate (13.56%), linalool (1.71%), *β*-caryophyllene (1.07%) and ocimene (0.27%) [25].

Up to a dose of 5000 mg/kg b.w., mice receiving oral administration of *C. martini* essential oils did not exhibit treatment-related mortality, and no symptoms of toxicity or morbidity were noticed. The results of the investigation on acute toxicity revealed that the LD_50_ of *C. martini* essential oil was more than 5000 mg/kg b.w. Based on this finding, the general harmonized system (GHS) of the United Nations has classified the essential oil of *C. martini* as belonging to the class of chemicals with a low acute oral toxicity, or the 5^th^ class of toxicity [26].

In line with the findings of Andrade et al., subacute treatment of essential *C. martini* at 500 mg/kg and 1000 mg/kg b.w had no discernible impact on the body weight and food intake of mice [25].

According to this study's evaluation, RBC, WBC, PC, HB, MCH, MCV, and MCHC did not substantially alter in mice following treatment with *C. martini* essential oil. This discovery has not yet been reported so far.

The release of cytosolic enzymes into the circulation happens in conjunction with plasma membrane damage from hepatic cells under various pathological circumstances. After exposure to harmful agents, the aminotransferases ALT and AST are utilized to diagnose liver damage [27]. Following therapy with *C. martini* essential oil, these parameters in mice did not alter appreciably. In this instance, Andrade et al. reported a similar outcome [25].

## 5. Conclusions

The essential oil of *C. martini* was shown to be neither acutely poisonous nor sub acutely harmful in mice. Using this oil for cosmetic reasons is safe, according to the information from the present study. Finally, it can be claimed that the essential oil of Ethiopian *C. martini* is nontoxic. Future research should be done to corroborate the current findings, and studies on long-term toxicity should be utilized to determine how safe it is for humans.

## Figures and Tables

**Table 1:** Changes in body weight of mice after treatment with 500 mg/kg and 1000 mg/kg b.w. of *C. martini* essential oil.

Treatment	Dose (mg/kg)	Weight (gram)			
		Week 1	Week 2	Week 3	Week 4
Control	10 ml/kg	29.6 ± 3.4	30.6 ± 3.6	31.3 ± 3.7	32.9 ± 2.1
CM	500	27.2 ± 4.8	28.4 ± 7.6	29.1 ± 6.9	26.8 ± 3.2
CM	1000	28.5 ± 3.5	26.8 ± 3.2	25.8 ± 4.2	24.8 ± 3.2
*P* value		0.07	0.16	0.38	0.59

**Table 2:** Changes in food consumption of mice following treatment with essential oil of *C. martini* at 500 mg/kg and 1000 mg/kg b.w.

Treatment	Dose (mg/kg)	Food consumption (gram)			
		Week 1	Week 2	Week 3	Week 4
Control	10 ml/kg	26.2 ± 0.9	26.5 ± 1.3	25.0 + 1.2	26.4 ± 0.6

CM	500	24.0 ± 1.7	24.2 ± 2.0	23.5 ± 1.4	24.0 ± 1.8
	1000	23.5 ± 2.6	23.0 ± 1.3	23.0 ± 2.3	22.0 ± 2.9

*P* value	—	0.08	0.23	0.17	0.25

**Table 3:** Effect of *C. martini* essential oil on hematological parameters in mice.

Treatment	Dose (mg/kg)	RBC (10^6^/mm^3^)	WBC (10^3^/mm^3^)	PC (10^5^/*μ*l)	HB (gm.)	MCH (g/dl)	MCV (g/dl)	MCHC (g/dl)
Control	10 ml/kg	9.79 ± 8.03	7.63 ± 2.50	11.15 ± 0.75	14.7 ± 0.5	34.54 ± 3.46	49 ± 5	32.5 ± 4.5
CM	500	9.15 ± 0.7	7.68 ± 2.80	10.45 ± 2.45	12.81 ± 0.99	34.74 ± 3.26	48.3 ± 5.7	34.8 ± 1.2
CM	1000	9.34 ± 0.45	7.79 ± 3.23	9.55 ± 3.45	13.3 ± 1.4	34.94 ± 3.06	48.8 ± 6.2	35.3 ± 2.7
*P* value		0.67	0.27	0.62	0.27	0.48	0.43	0.86

**Table 4:** Effect of essential oil of *C. martini* on biochemical parameters in mice.

Treatment	Dose (mg/kg)	AST (U/L)	ALT (U/L)	ALP (U/L)	Total chol. (gm)	BUN (mmol/L)		Creatinine (mg/dl)
Control	10 ml/kg	74.7 ± 7.3	45.5 ± 12.5	67.2 ± 18.8	52.4 ± 17.6	22.8 ± 4.2	0.125 ± 0.015	
CM	500	72.8 ± 23.2	49.7 ± 10.7	68 ± 14	54.2 ± 9.80	25.4 ± 3.26	0.128 ± 0.01	
CM	1000	71.16 ± 7.4	43.7 ± 13.2	62.5 ± 7.45	50.4 ± 20.6	23.6 ± 3.06	0.116 ± 0.05	
*P* value		0.27	0.27	0.85	0.56	0.50	0.10	

## Data Availability

The majority of the data are included in this manuscript. Further data can be found from the corresponding author based on reasonable request.

## Abbreviations

ALT: Alanine transaminase
ALP: Alkaline phosphatase
AST: Aspartate transaminase
BUN: Blood urea nitrogen
LD_50_: Median lethal dose
MCH: Mean cell hemoglobin
MCHC: Mean cell hemoglobin concentration
MCV: Mean cell volume
OECD: Organization for Economic Cooperation and Development
PC: Platelet count
RBC: Red blood cell count
WBC: White blood cell count.
